# The impact of spinal fusion of adolescent idiopathic scoliosis in Salah (Islamic Prayer) movement: a case-control study

**DOI:** 10.12688/f1000research.124255.1

**Published:** 2022-09-15

**Authors:** Komang Agung Irianto, Naufal Ranadi Firas, Carlos Gracia Supriantono Binti, Damayanti Tinduh, Yudha Mathan Sakti, Brigita De Vega

**Affiliations:** 1 Department of Orthopedic and Traumatology, Faculty of Medicine, Universitas Airlangga/ Dr. Soetomo General Academic Hospital, Surabaya, East Java, 60286, Indonesia; 2 Surabaya Orthopedic Traumatology Hospital, Surabaya, East Java, 60213, Indonesia; 3 Department of Physical Medicine and Medical Rehabilitation, Faculty of Medicine, Universitas Airlangga/ Dr.Soetomo General Academic Hospital, Surabaya, East Java, 60286, Indonesia; 4 Department of Orthopedic and Traumatology, Faculty of Medicine, Public Health, and Nursing, Universitas Gadjah Mada/ Dr. Sardjito General Hospital, Sleman, Yogyakarta Special Region, 55281, Indonesia; 5 Institute of Orthopaedics and Musculoskeletal Science, Division of Surgery and Interventional Science, University College London, London, NW3 2PS, UK

**Keywords:** Adolescent, Scoliosis, Adolescent Idiopathic Scoliosis, Spine, Spinal Fusion, Islam

## Abstract

Background: Corrective spine surgery is widely accepted for treating severe adolescent idiopathic scoliosis (AIS). Postoperative spinal range of motion (ROM) could be affected after such surgery. In certain populations, such as Muslims, this ROM change can impact daily life, as it may affect the five-times-a-day prayer (Salah). This study aims to assess the influence of spinal fusion (SF) in Adolescent Idiopathic Scoliosis (AIS) during the daily Islamic prayer (Salah).

Methods: SF-AIS patients were videoed while performing Salah prayer. The kinematic documentation was assessed and compared to Salah movements of a control group of age-matched Muslim AIS patients, who had not had surgery. The prayer quality changes were subjectively classified into improved, no change/remained, and worsened, according to the Global Perceived Effect (GPE). Functional outcome and pain were assessed by the Scoliosis Research Society Questionnaire Version 30 (SRS-30).

Results: Thirty-nine women and five men (mean age±SD: 14.8±2.3 years) met the inclusion criteria, and unoperated AIS patients were used as control (twenty-two women, mean age±SD: 15.32±1.43 years). The prostrations ROM of the SF-AIS group differed significantly from the control group (p<0.05). The GPE of the prayer movement showed improvement in 36.4%, no change in 59.1%, and worsening in 4.5% of the SF-AIS patients. The worsened group had a significantly lower bowing ROM and higher prostrations ROM compared to all groups of prayer quality changes (p<0.05). SRS-30 scores showed good outcomes (function 4.0±0.2, pain 4.2±0.5), along with the overall bowing ROM and prostrations ROM (84.2±12.0° and 53.4±9.6°, respectively). Moreover, a significant moderate positive correlation between the bowing ROM and pain (r=0.417, p=0.007) was also found.

Conclusion: Spinal fusion positively affects AIS Islamic patients in maintaining their daily Salah movement, ROM and prayer quality. Prayer quality assessment should be given extra attention as an adjuvant of the SRS-30 questionnaire to evaluate Muslim patients.

## Introduction

Corrective spine surgery is widely accepted to improve the overall quality of life (QOL) for adolescent idiopathic scoliosis (AIS). Maximal deformity correction in achieving the coronal and sagittal balance while retaining the spine flexibility will attain the desired cosmetic look and potentially bring back patient’s lost self-esteem. ^1^^,^^2^ A study by Weiss and Goodall, discovered that patients’ ability to perform flexion was reduced by 20–60% following scoliosis correction surgery. ^3^ Meanwhile, physical impairment in any disease corresponds to physiological abnormalities. In AIS, self-esteem is one of the most important aspects that build the patient’s courage to endure a serious high-risk spinal surgery. Likewise, physical function is an important outcome that could portray a meaningful individual’s QOL. ^4^

Interestingly, pain is not the primary reason for patients seeking treatment. Patients can move and perform daily activities without pain before the surgery. Disability, defined as the diminished capacity for everyday activities and gainful employment, is a common cause for a patient seeking medical attention. Nevertheless, pain and disability assessments are highly personal; thus, they may differ among individuals. ^5^^,^^6^ Patients also demand information regarding the continuation and possible improvement following corrective surgery, which this study emphasized in performing daily prayer (Salah) for Muslims.

For decades, the AIS surgical treatment has encompassed pedicle screw fixation to fuse vertebral segments while maintaining growth preservation in the immature AIS. However, the older Harrington instrumentation technique has a higher risk of loose correction. ^1^ Studer *et al*., studied 157 AIS patients who underwent spinal fusion surgery. Although they reported minimal complications with no revision surgery, they did not relate to the outcome of QOL. Therefore, an additional evaluation of clinical and patients’ health-related QOL as a reliable outcome is needed rather than solely radiologic or curve measurement. ^5^

Furthermore, Helenius *et al*., learned that in the five years of follow-up, scoliosis correction surgery in AIS could reduce back pain and improve QOL compared to untreated AIS patients. The QOL outcome was similar to healthy patients. ^1^ Therefore, utilizing an objective assessment to measure functional outcomes is essential, in addition to subjective questionnaires. Scoliosis Research Society Questionnaire Version 30 (SRS-30) has been used in many studies to objectively measure the functional outcome of scoliosis patients. ^6^^,^^7^ The questionnaire contains 30 questions covering five domains, including 1) function/activity, 2) pain, 3) self-image/appearance, 4) mental health, and 5) satisfaction with management. Nevertheless, existing tools used to assess post-surgery impacts on the activities of daily living (ADL) could not portray an important activity for Muslim patients, for instance, practicing five-times-a-day prayer. In Indonesia, the largest Muslim-populated country, it is essential to have AIS patients fully informed about the possible impact of the post-surgery spinal fusion on their Salah (Islamic prayer).

Islamic prayers consist of a certain number of Rak’ah, which involves several repetitive movements and postures. Every day, Muslims are obligated to perform 119 postures from 7 to 10 years old. ^8^^,^^9^ These gave numerous physical and physiological benefits since almost all body muscles and joints are exercised during prayers. ^9^^–^^11^ The Salah movements are composed of standing upright, bowing, prostration, and sitting with knees bent; these movements need maximum extension and flexion of the spine. ^10^^,^^11^ The flexors and extensors of weight-bearing joints are also involved during various Salah positions. ^10^^,^^11^ Islamic prayer has a similar effect as gentle exercises that cause muscles to contract isometrically and isotonically. These movements are beneficial in maintaining joint mobility and elasticity of its surrounding structures. ^9^^,^^10^ Although Salah involves continuous gentle muscle contraction and relaxation with perfect harmony and balance, it may be modified depending on certain medical conditions. ^8^^,^^9^ AIS patients expect their ADL (including the compulsory prayers for Muslims) to be unaffected following the spinal fusion corrective surgery. To the best of our knowledge, the evaluation of Islamic prayer (Salah) movements in scoliotic patients and the possible impact of corrective spinal fusion on practicing Salah movements has not yet been studied. Therefore, this study aims to primarily assess the influence of spinal fusion (SF) in AIS during the daily Islamic prayer (Salah) by assessing the Salah prayer quality changes following spinal fusion surgery. By comparing the postoperative Salah movement ROM to age-matched Muslim unoperated AIS patients, this study aims to identify whether spinal fusion surgery affects the Muslims’ quality of life (including their prayer quality).

## Methods

### Ethics

Ethical clearance was approved by the Ethical Review Committee of Faculty of Medicine, Airlangga University (institutional review board approval no. 285/EC/KEPK/FKUA/2020). The research team obtained written informed consent from participants before study commencement. All participants consented to be video recorded and photographed during one Salah movement. The participants also consented to having their videos/images and data (excluding name/contact details) published.

### Setting, patient selection, eligibility criteria, and surgical technique

This case-control study was conducted in three orthopedic centers in Surabaya and Yogyakarta, Indonesia, by retrieving data from physical and electronic medical records from 2010 to 2020. The three orthopedic centers were chosen due to their roles as the main referral centers and prime affiliated teaching hospitals in the region. Four research team members (KAI, NRF, CGS, and YMS) screened patients’ data for their eligibility with a predefined form (containing the patient’s personal information such as name and phone number, age during surgery, sex, religion, medical history, pre-operative Cobb angle, post-operative Cobb angle, fusion level, Lenke classification, and follow-up duration). No additional authorization (in addition to the ethical clearance) was required to access patients’ records because the researchers involved in data collection were the relevant orthopedic surgeons in the respective centers. Patients who met the inclusion criteria were telephoned by the authorized research team members (KAI, NRF, CGS, and YMS) to be invited to the research program.

The inclusion criteria for this study were: 1) patients suffering from AIS alone (without any neuromuscular deficit), who underwent spinal fusion corrective surgery; 2) completed at least two years of follow-up post-surgery; 3) are Muslim; 4) consented to be video recorded during one Salah movement. The surgery was offered by all three orthopedic centers using freehand technique and fluoroscopy assistance for final confirmation. Posterior spinal fusion surgery was conducted by bilateral segmental pedicle screw instrumentation following a combination of vertebral column derotation and selective translation, compression, and distraction maneuver.

### Outcome data collection and assessment

**Figure 1.**
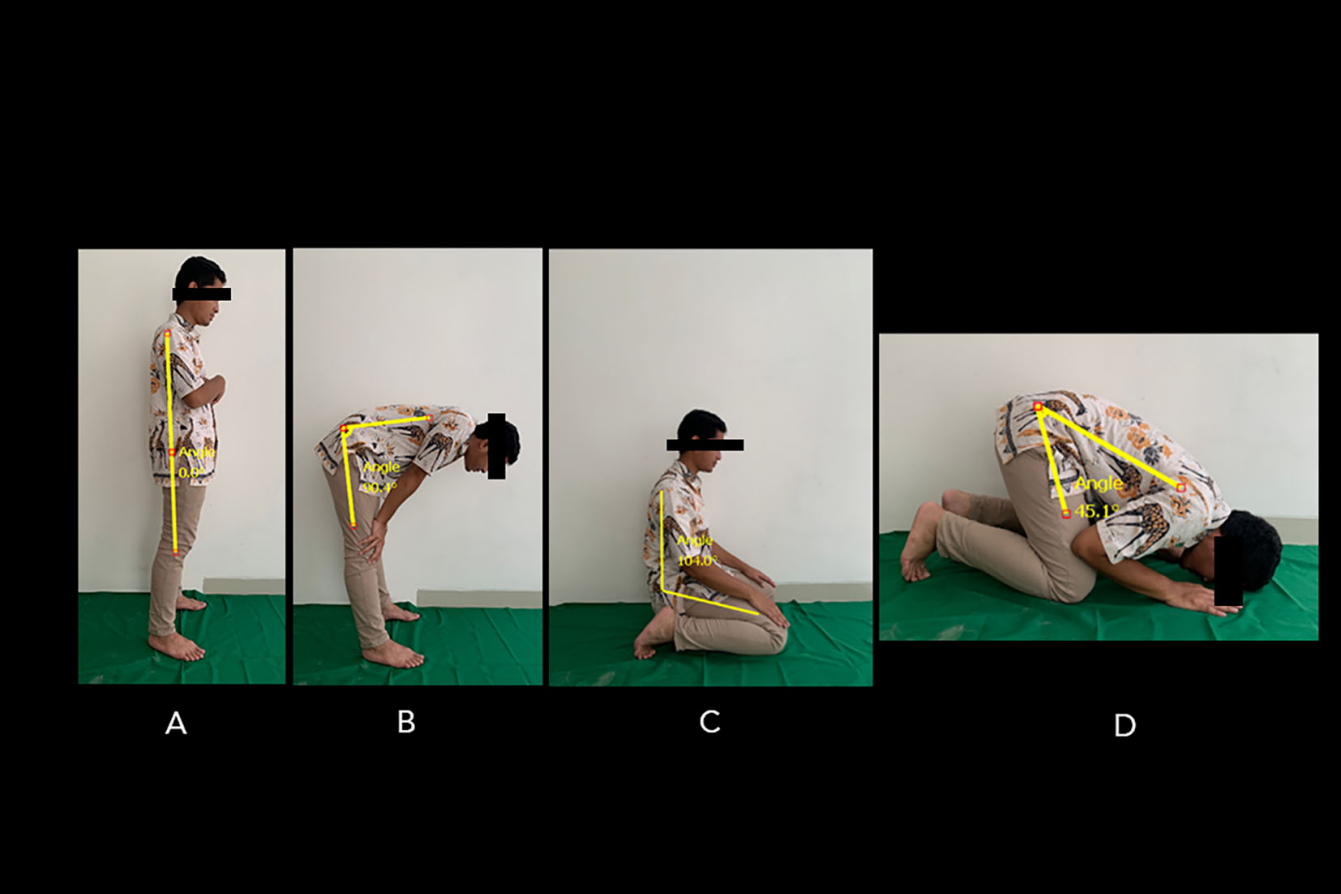
Standard movements’ series of Muslim prayers. The position of the Salah movement involves: (A) standing position, (B) bowing, (C) sitting between prostrations, and (D) prostration. The ROM of standing-to-bowing movement was measured by obtaining the angle difference between A and B. The ROM of the sitting position between two prostrations to prostration was measured by obtaining the angle difference between C and D. Image source: authors.

**Figure 2.**
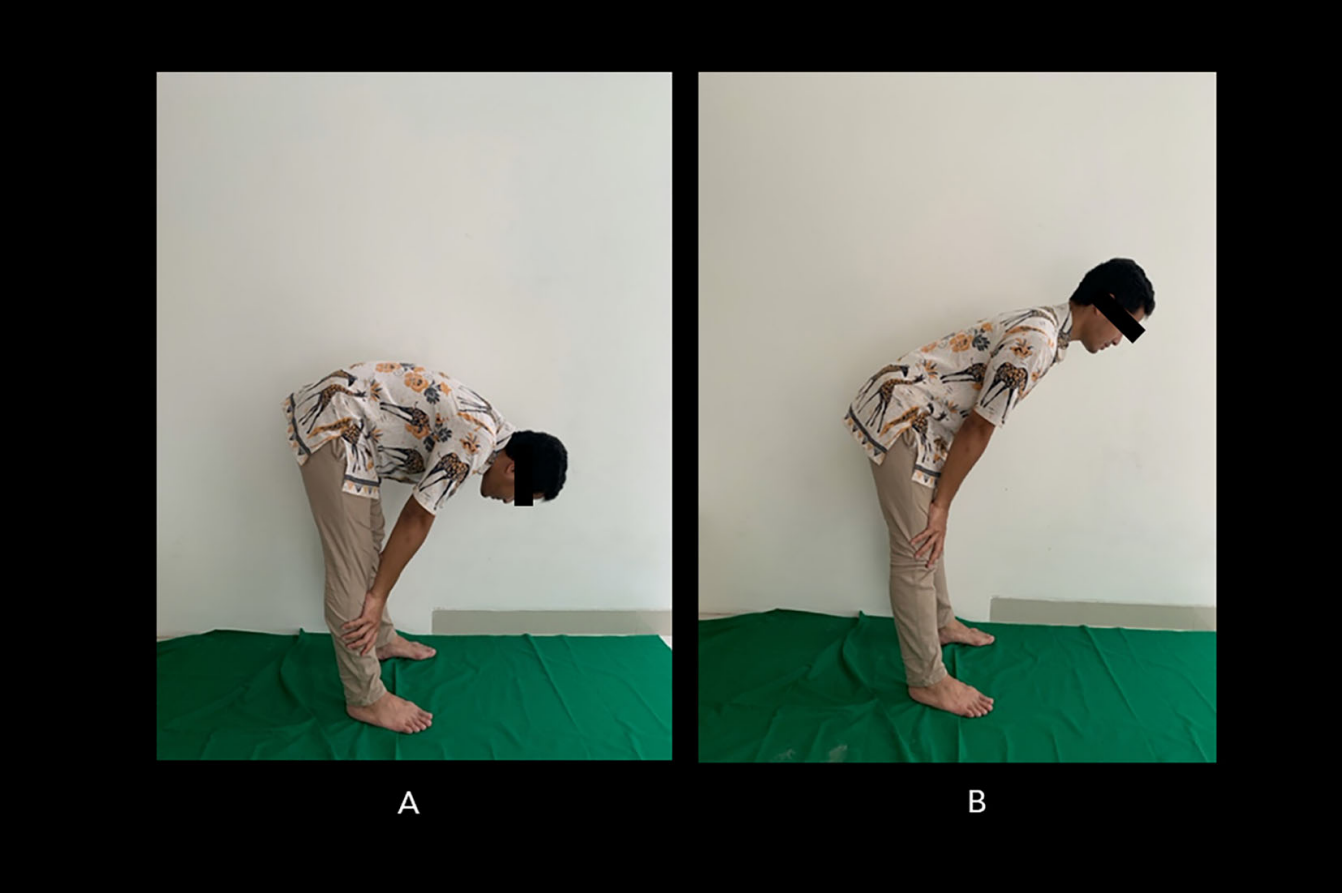
Incorrect bowing (ruku’) movements during Muslim prayer. (A) The back is overly bent; (B) the back is too upright. Image source: authors.

Eligible patients were invited to the outpatient clinic in all three orthopedic centers and asked to perform one Salah movement, during which they were videoed and photographed by orthopedic residents. The range of movement (ROM) during the Salah were evaluated and analyzed via video recordings and photos using the Kinovea software application (version 0.8.15). ^12^ This tool can track computed points, distances (up to 5m distance from an object), and measure angles. The Salah’s movement consisted of four postures ( Figure 1). ^8^^,^^9^ The first and second postures, namely standing upright (Qayyam) and bowing (Ruku), comprise a series of movements involving: the standing position ( Figure 1A) and bowing (approximately 90 degrees), while the fingers are stretched with two knees clasped firmly, elbows are extended and facing inwards, back is flattened, and the head is not tilted nor bent (i.e., parallel to the back) ( Figure 1B). Next, sitting with knees bent (Taashahhud) is performed by kneeling and sitting on the top of the left foot while the right foot rests on the toes. The toes should face forward towards the Qibla (the direction in which the Muslims are praying) ( Figure 1C). Finally, prostration (Sajdah) places both palms, knees, feet, forehead, and nose on the floor ( Figure 1D). The ROM of standing-to-bow (i.e., bowing ROM) movement was measured by obtaining the angle difference between Figure 1A and Figure 1B. The ROM of the sitting position between two prostrations to prostration (i.e., prostrations ROM) was measured by obtaining the angle difference between Figure 1C and Figure 1D. Figure 2 shows the incorrect Salah movement.

The patients were then asked to fill out the SRS-30 questionnaire as an assessment for their QOL. Among five assessment categories in the questionnaire (pain, function or activity, self-image, mental health, and satisfaction with management), we only utilized two of them (pain and function or activity) as these are the most relevant assessment categories to our study. The score ranges from 1 to 5, with a higher SRS score showing a better outcome. The patients were also categorized based on their subjective perception of their prayer quality changes, which were classified into 1) Improved, 2) No change/remained, and 3) Worsened. This subjective perception is based on global perceived effect (GPE), a commonly used tool to assess patients’ own viewpoint on how much their condition has improved or deteriorated since a particular time point in interest. ^13^ In the present study, the GPE was used to assess the overall situation concerning their prayer movement compared to pre-surgery conditions. To acquire an objective comparison, 22 age-matched Muslim unoperated AIS patients were recruited as a control group.

### Data analysis

We analyzed several outcomes, such as postoperative SRS-30 (pain and functional outcome) and postoperative Salah prayer ROM (bowing and prostrations) of the SF-AIS patients, which were compared to the control group. The patients in the intervention (operated) group were grouped based on their prayer quality changes perception/GPE (improved, no change/remained, worsened) and compared. We also conducted a correlation analysis to identify whether the outcomes (including some variables such as Cobb angle correction and total fused level) affect one another.

All statistical analyses were performed using SPSS software version 23.0 (IBM, Chicago, USA). The normality test was performed using the Shapiro-Wilk test. ^14^ Discrete data were presented in frequency and percentage (%), while continuous data were presented in mean and standard deviation (mean±SD). When the data were normally distributed, the outcome comparison among the groups was analyzed using the ANOVA parametric test. In contrast, the non-normally distributed data were analyzed using the non-parametric Kruskal-Wallis test. ^14^ Differences between group means were compared using appropriate Post Hoc tests (Post hoc Games-Howell test following ANOVA, post hoc Mann-Whitney test following Kruskal Wallis). ^15^ The correlation analysis among the outcomes was calculated using the Spearman rank test. ^14^ A p-value of <0.05 was considered to be statistically significant.

## Results

### Patient characteristics

Forty-four SF-AIS Muslim patients met the inclusion criteria, consisting of 39 women (88.6%) and five men (11.4%). Moreover, unoperated AIS patients were included in the control group (22 women). The summary of demographics, characteristics, and outcomes of the AIS patients is presented in Table 1. In contrast, a detailed description of the included patients is provided in *Extended data.* ^40^ Overall, the average SF-AIS patients’ age at surgery was 14.8±2.3 years (range 10-18 years), with an average follow-up time of 4.4±1.9 (range 2-9) years.

**Table 1.** Demographics, characteristics, and outcomes of the AIS patients included in the study *.

Variable	Mean±SD or frequency (%)	
	Intervention (operated) group	Control (unoperated) group
Age ^a^	14.8±2.3 years	15.3±1.4 years
Sex ^b^	Men=5 patients (11.4%) Women=39 patients (88.6%)	Women=22 patients (100%)
Follow-up duration	4.4±1.9 years	N/A
Preoperative Cobb angle ^c^	67.1±16.2 ^o^	52.3±14.0 ^o^
Postoperative Cobb angle	32.3±12.9 ^o^	N/A
Cobb angle correction	34.8±12.8 ^o^	N/A
Total fused level	11.4±2.8	N/A
Lowest fused level	L1 and above=5 patients (11.4%) L2=9 patients (20.5%) L3=14 patients (31.8%) L4=12 patients (27.3%) L5 and below=4 patients (9.1%)	N/A
Prayer quality changes (GPE)	Improved=16 patients (36.4%) No change/remained=26 patients (59.1%) Worsened=2 patients (4.5%)	N/A
SRS 30 Function	4.0±0.2	N/A
SRS 30 Pain	4.2±0.5	N/A
Bowing ROM	84.2±12.0 ^o^	87.8±9.3 ^o^
Prostrations ROM	53.4±9.6 ^o^	58.5±4.7 ^o^

N/A: Not applicable.

* Detailed description is available in Appendix 1 and 2.

^a^ Mann-Whitney test p=0.466.

^b^ Fisher’s exact test p=0.160.

^c^ Independent t-test p=0.000

Similarly, the average age of the control group was 15.32±1.43 (range 13-18) years. Although the preoperative Cobb angles between the two groups differed significantly (67.1±16.2° and 52.3±14.0°, respectively), it does not necessarily reflect a non-homogeneity between them. The nature of this study is to compare the operated and unoperated patients. Those who underwent surgery would generally have a higher degree of Cobb angle, though the clinical significance of 14.9° mean difference is debatable. Moreover, the mean Cobb angle correction of the intervention (operated) group was 34.8±12.8°, with an average total fused level of 11.4±2.8. The highest spinal fusion level was T1, and the lowest was L5, with L3 being the most frequent lowest fused level (31.8%).

### Outcome description

**Figure 3.**
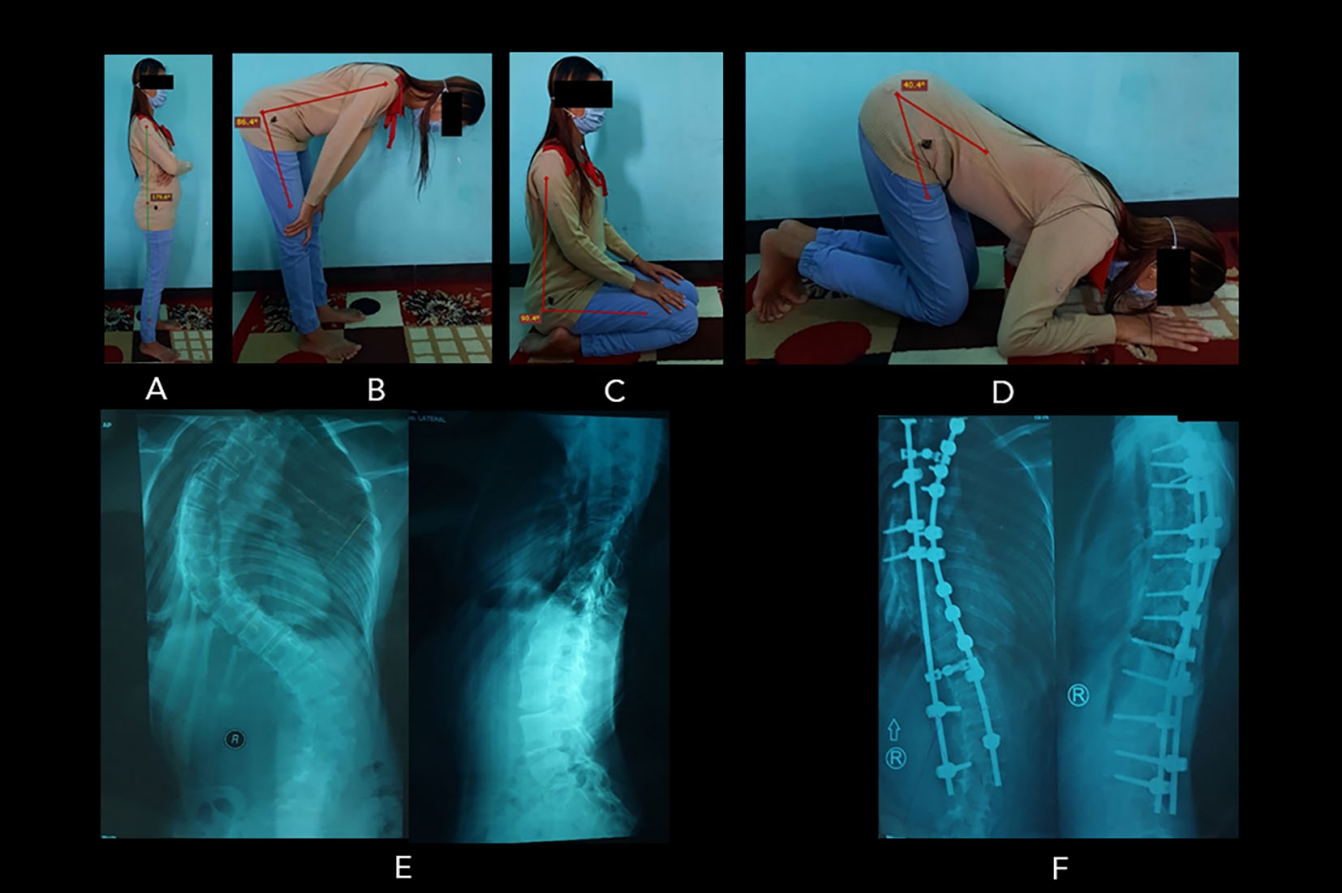
Patient number 6 (20-year-old woman, surgery was at 14 years old) experienced an improvement in the quality of prayer. ROM measurement of the prayer movement: (A) standing position (179.6 ^o^); (B) bowing position (86.4 ^o^); (C) sitting between prostrations (90.4 ^o^); to (D) prostration (40.4 ^o^). The patient’s radiographs showed the preoperative (E) and postoperative (F) conditions of the spine. In these radiographs, the preoperative Cobb angle was 50 ^o^, and the postoperative Cobb angle was 18 ^o^ (Cobb angle difference: 32 ^o^). Image source: authors.

Despite the overall relatively good postoperative SRS-30 score (4.0±0.2 for function and 4.2±0.5 for pain), the quality of prayer movement, which was subjectively expressed by the patients as a global perceived effect (GPE), improved only in fifteen women and one man (36.4%) ( Table 1). Figure 3 shows the example of Salah movements from a patient who expressed an improved prayer quality following surgery. Whereas the majority of the patients (22 women and four men) reported the same prayer quality compared to preoperative condition (no changes/remained) (59.1%). Two women (4.5%) felt that their prayer movement worsened due to the difficulties in bowing and bending properly. Interestingly, the SRS-30 scores of both worsened patients were reasonably good, i.e., 3.7 and 4.1 for functional outcome and 3.2 and 4 for pain (maximum score of 5) (see *Extended data*).

**Figure 4.**
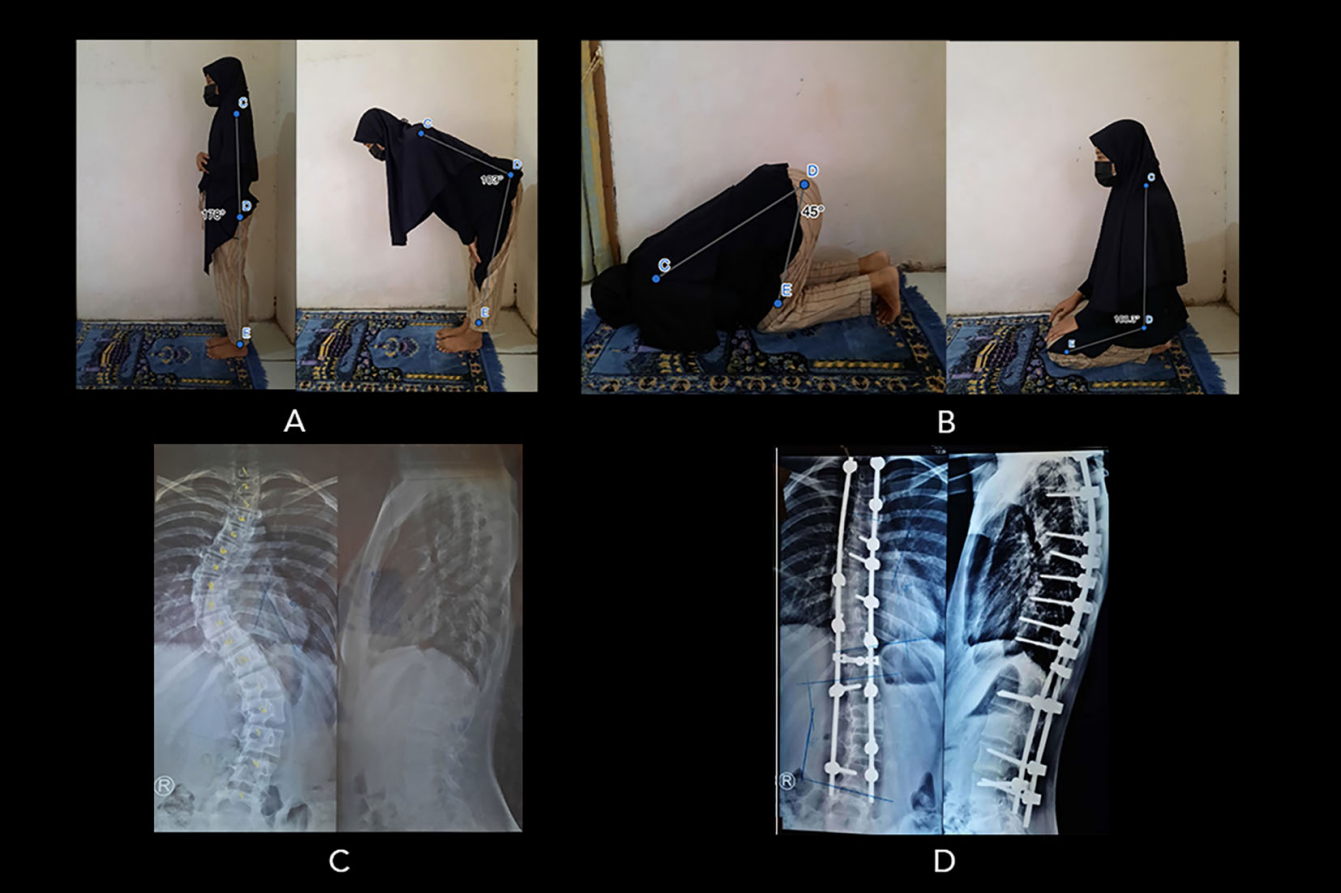
Patient number 43 (16-year-old woman, surgery at 13 years old) complained of worsening prayer quality following surgery. (A) The patient’s bowing movement ROM was within the normal range: (178 ^o^ to 73 ^o^), but she experienced a worsened prayer quality because of pain. (B) She can perform sitting motion between two prostrations and prostration as good as normal (108 ^o^ to 45 ^o^) but accompanied by pain. (C) Preoperative radiograph showing Cobb angle 45 ^o^. (D) Postoperative radiograph showing Cobb angle 20 ^o^ (Cobb angle difference: 25 ^o^). Image source: authors.

The mean postoperative bowing ROM in SF-AIS patients was lower than the control group (84.2±12.0°, range 26-101° vs. 87.8±9.3°, range 71.5-106.4°, respectively) ( Table 2 and *Extended data*). One SF-AIS patient (in the “worsened” group) had lower bowing ROM than the control group. Moreover, the mean prostrations ROM in SF-AIS patients was also lower (53.4±9.6°, range 39.6-100°) compared to the control group (58.5±4.7°, range 49.2-66.4°). Twelve SF-AIS patients (five in the “improved” group, seven in the “no change” group) had lower prostrations ROM compared to controls. Interestingly, there was one SF-AIS patient who complained of an overall “worsened” prayer quality (Patient 43), but their bowing and prostrations ROM were within the normal range ( Figure 4). Moreover, the other patient who also reported “worsened” prayer quality (Patient 44) had a strictly limited bowing ROM but excessive prostrations ROM instead (26° and 100°, respectively) (see *Extended data*). None of the operated patients had revision surgery or complication.

**Table 2.** Bowing and Prostration ROM in intervention vs. control group.

Variable	Intervention group	Control group	p-value
Bowing ROM	84.2±12.0	87.8±9.3	0.366
Prostrations ROM	53.4±9.6	58.5±4.7	0.000 *

Note: Analyzed using Mann-Whitney non-parametric test. P-values of the tests are shown.

* Statistically significant (p<0.05).

### Outcome analysis

We compared bowing and prostration ROM between SF-AIS patients and the control group ( Table 2) and the outcomes across the three GPE prayer quality (improved, no change/remained, worsened) groups ( Table 3). The prostrations ROM of the SF-AIS group differed significantly from the control group (p=0.000). However, the bowing ROM differences between the SF-AIS and control groups were insignificant (p=0.366). Although the Cobb angle correction was lower and the total fused levels were higher in the “worsened” group than the “improved” and “remained” group, their differences were insignificant (p=0.678 and 0.115, respectively). Similarly, the “worsened” group showed worse SRS-30 function and pain than the other two groups, but the differences were insignificant (p=0.253 and 0.700, respectively).

**Table 3.** Comparison of the outcomes across three groups of prayer quality changes (intervention group).

Variable	Prayer Quality Changes (GPE)			p-value
	Improved (n=16)	No change/remained (n=26)	Worsened (n=2)	
Cobb angle correction ^a^	33.9±11.2 ^o^	35.8±14.1 ^o^	28.0±4.2 ^o^	0.678
Total fused level	11.4±2.1	11.1±3.1	15.0±1.4	0.115
SRS 30 Function	4.1±0.5	4.2±0.5	3.6±0.6	0.253
SRS 30 Pain	4.0±0.3	4.1±0.1	3.9±0.3	0.700
Bowing ROM	84.7±9.0	86.8±6.3	45.5±27.6	0.042 *
Prostrations ROM	51.4±5.5	52.1±5.8	86.7±18.9	0.040 *

Note: Analyzed using Kruskal-Wallis non-parametric test, unless stated otherwise.

^a^ Analyzed using ANOVA.

* Statistically significant (p<0.05).

In general, the “remained” group had the highest bowing ROM while the “worsened” group had the lowest (50.5±34.6°) ( Table 3). Surprisingly, the “worsened” group showed the highest prostrations ROM compared to the other groups of prayer quality changes (as high as 81.7±26.0°), with a significant mean difference (p=0.040). The pairwise comparison post hoc tests ( Tables 4 and 5) showed that the “worsened” group had a significantly lower bowing ROM and higher prostrations ROM compared to all groups of prayer quality changes.

**Table 4.** Post hoc analysis of bowing ROM means across three groups of prayer quality changes (intervention group).

Group	Improved	No change/remained	Worsened
Improved	N/A	0.364	0.024 *
No change/remained	0.364	N/A	0.02 *
Worsened	0.024 *	0.02 *	N/A

Note: Mann-Whitney test was used for post hoc analysis. P-values of the tests are shown.

* Statistically significant (p<0.05).

**Table 5.** Post hoc analysis of prostration ROM means across three groups of prayer quality changes (intervention group).

Group	Improved	No change/remained	Worsened
Improved	N/A	0.337	0.024 *
No change/remained	0.337	N/A	0.02 *
Worsened	0.024 *	0.02 *	N/A

Note: Mann-Whitney test was used for post hoc analysis. P-values of the tests are shown.

* Statistically significant (p<0.05).

The correlation analyses ( Table 6) showed that the Cobb angle correction and total fused levels did not correlate with either the quality of life (SRS-30 function and pain) or Salah ROM (bowing and prostrations ROM). Likewise, we found that SRS-30 function and pain did not correlate significantly with Salah ROM, except for bowing ROM. Our study showed a significant moderate positive correlation between bowing ROM and pain score (r=0.401, p=0.007).

**Table 6.** Correlation analysis among the outcomes (intervention group).

Variable	Cobb angle correction	Total fused level	SRS-30 Function	SRS-30 Pain	Bowing ROM	Prostrations ROM
Cobb angle correction	N/A	N/A	r=0.113 p=0.467	r=-0.245 p=0.109	r=0.000 p=0.997	r=-0.083 p=0.590
Total fused level	N/A	N/A	r=-0.073 p=0.639	r=0.013 p=0.932	r=0.178 p=0.248	r=-0.243 p=0.111
SRS-30 Function	r=0.113 p=0.467	r=-0.073 p=0.639	N/A	N/A	r=0.123 p=0.425	r=0.138 p=0.373
SRS-30 Pain	r=-0.245 p=0.109	r=0.013 p=0.932	N/A	N/A	r=0.401 p=0.007 *	r=0.070 p=0.652
Bowing ROM	r=0.000 p=0.997	r=0.178 p=0.248	r=0.123 p=0.425	r=0.401 p=0.007 *	N/A	N/A
Prostrations ROM	r=-0.083 p=0.590	r=-0.243 p=0.111	r=0.138 p=0.373	r=0.070 p=0.652	N/A	N/A

* Statistically significant (p<0.05).

## Discussion

Posterior spinal fusion is a primary surgical treatment for adolescent idiopathic scoliosis, which led to QOL improvement. ^1^^,^^3^^,^^5^^,^^12^^,^^13^ A Bayesian meta-analysis study on the effectiveness and safety of surgical interventions for treating AIS revealed that posterior spinal fusion is a primary surgical treatment due to the lower complication rate. ^16^ Our present study showed surgical outcomes following single-stage reconstruction to objectively measure values for the quality of Islamic prayer (Salah) range of motion (ROM) following spinal fusion in adolescent idiopathic scoliosis (AIS). The world’s Muslim population as of 2021 is estimated as high as over 1.9 billion people, making 24.7% of the world’s total population today. ^17^ This number is projected to increase at a rate of 1.5% annually. By 2030, a study approximated that Muslims will make 26.4% (2.2 billion people) of the world’s total population. ^18^ Muslims inhabit almost all countries (over 70 countries) globally, with Asia-Pacific and Africa being the largest populated regions (60% and 35%, respectively). As the country with the single largest Muslim population, Indonesia is home to over 242 million Muslims (making over 87% total population of the country and 12.5% world’s Muslim population) in 2021. ^18^^,^^19^ In Indonesia, the scoliosis prevalence in secondary school screening was 2.9%. ^20^ Indeed, most Indonesian AIS patients are presumably Muslims. Thus, they will need all the information about the postoperative outcomes for their lifelong quality of life. However, the quality of Islamic prayer practiced regularly (five times a day) concerning spinal ROM as a crucial part of practicing the whole serial movement is not covered by all generic surgical outcome evaluations for health-related QOL.

### The impact of spinal fusion surgery on Salah prayer quality and ROM

In general, we found that spinal fusion surgery did not negatively affect the Salah prayer quality, as 95.5% of the operated patients reported improved or similar prayer quality as the preoperative condition. The quality of life, including daily prayers five times a day, could improve after the surgery. Although the ROM improvement may only be modest, spinal fusion surgery still enhances patients’ quality of life. ^21^ Only two patients (4.5%) reported a worsened prayer quality following surgery, one of whom showed a somewhat extreme value of bowing and prostrations ROM (26° and 100°, respectively). There are several possible factors contributing to this phenomenon.

Firstly, despite longer fused levels (15.0±1.4), the Cobb angle correction in the “worsened” group is less than in the other groups (28.0±4.2°), which might partly explain the restricted bowing ROM. A study by Cho *et al*., reported that longer fused levels were associated with significantly better Cobb angle correction than shorter fused levels (72% and 39%, respectively, p=0.001). ^22^ Secondly, the excessive prostration ROM is probably due to the hyperextended lumbar condition. Longer fused levels could restore lumbar lordosis better; however, hypercorrection might lead to lumbar hyperextension. ^23^ Thirdly, the patient could only make a sitting position between the two prostrations and could not proceed to the prostration movement. Thus, the prostration ROM might have seemed greater.

During the bowing movement, one should maintain the lower back flexed, followed by resting the forehead gently on the floor (i.e., prostration movement), activating postural neck muscles to control the neutral head position when lowering down and lifting it from the floor. ^11^ The stretch felt along the spine as the individual curls the torso over the legs also creates a space between the dorsal surfaces of the vertebra, aiding spinal distraction, allowing neural glides and nerves lengthening. ^11^ Our findings showed insignificant differences (p≥0.05) in bowing ROM between treated AIS patients in comparison to the controls but not prostrations ROM ( Table 2), implying that bowing ROM following surgery is comparable to the unoperated AIS patients. This distinct finding might be caused by the difference in muscles used during the two movements. Ruku’ (bowing) uses neck extensors (NE), deltoid (DT), triceps brachii (TB), and rectus abdominal (RA) muscles; in contrast, Sajdah (prostration) uses sternocleidomastoideus (SCM), trapezius (TRP), biceps brachii (BB), and erector spinae (ES) muscles. ^21^ Since the ES and other paraspinal muscles are retracted for long hours during surgery, ischemia may occur and cause degeneration. ^24^ Nonetheless, further studies might be needed to thoroughly understand the differences in each specific muscle used in Ruku’ and Sajdah to understand the effect on muscle after spinal fusion surgery.

Interestingly, we obtained a positive correlation between the bowing ROM and pain ( Table 6). During Ruku’s (bowing) movement, the surgical wound along the spinal axis will be stretched, causing pain and restricted motion. This finding needs to be informed to the AIS Muslim patients because their prayers and QOL might be affected due to pain. Moreover, Bastrom *et al*., who investigated the prevalence of postoperative pain in AIS and its association with preoperative pain, reported a 7% prevalence of unexplained postoperative pain within two years of follow-up. The unexplained pain was significantly correlated with preoperative pain. ^25^ Likewise, other studies have confirmed that increased baseline pain and psychological factors such as anxiety and helplessness are significant factors contributing to persistent pain (months to years) in 36-41.8% of SF-AIS patients, although gene expression HLA-DRB3 and surgery duration may also play a role. ^26^^–^^28^ Altogether, proper patient education is required since patients expect less pain and return-to-normal activities after corrective surgery.

### Prayer quality assessment as an adjuvant of QOL measurement using SRS-30 in Muslim patients

The results of this study showed that the scoliosis surgical outcome indicators (SRS-30; function and pain domain) in the three orthopedic centers were within good scores for all patients. The functional outcome and pain after posterior spinal fusion in our study were comparable to the SRS score reported by existing literature. ^7^ The SRS-30 questionnaire score for the normal population ranged from 4.1–4.6, while our patients’ score was 4.0±0.2 for function and 4.2±0.5 for pain. However, the GPE, which values the patients’ perception about the quality of their prayer’s movement, seemed to be not affected by SRS-30. Although the patients who reported a worsened prayer quality showed an overall lower SRS-30 score, the differences were insignificant ( Table 3). This finding could imply that the SRS-30 score (especially function and pain domain) might have different aspects for particular daily activities in a certain population. The quality of prayer has not been reflected in the SRS questionnaire, which warrants further consideration for orthopedic surgeons in explaining to Muslim scoliotic patients.

Patient number 44, who complained of pain in prostration posture while praying, scored 4 for pain, and the ROM was very limited compared to normal control. Patient 43 scored 3.2 for pain; nonetheless, the ROM for praying was within the normal range ( Figure 4). This was possibly due to her determination to pray as perfectly as possible to gain “normal” ROM, reflecting her coping mechanism. Several studies have reported that spinal fused-AIS patients (SF-AIS) have adapted to the fused condition to maintain the demanded posture in performing particular physical activities. ^29^^,^^30^ A case-control study by Kakar *et al*., which investigated the kinematics of the spine and lower extremity during high-effort running, found that SF-AIS patients expressed a significantly excessive lower trunk (by 6.1°) and pelvis (by 6.3°) segmental axial rotation while running compared to healthy controls but reduced ankle plantarflexion (by 9.2°) in the support phase; implying their compensatory mechanisms are possibly due to increased lumbar muscle stiffness and reduced proprioception. ^29^ Another study by Holewijn *et al*., who performed gait analysis on SF-AIS at increasing walking speeds (0.45 to 2.22 m/s), revealed that transverse plane thoracic-pelvic ROM was significantly diminished following spinal fusion surgery, with higher walking speeds showing more obvious differences. However, the lower body ROM, step length, and cadence remain unaffected, and SF-AIS patients could still walk with somewhat unaltered spatiotemporal parameters. ^30^

Many studies about the lumbar stiffness as the side effect of the spinal fusion in AIS have reported that the frontal plane of thorax-pelvis mobility improved, the volitional weight shifting that gave them postural control also improved, and the SRS score did not correlate with the outcome motion of the fused level. ^31^^–^^33^ However, these phenomena could not be covered by SRS-30 and its variants. The evaluation of scoliosis treatment comprises the surgical aspects, radiology aspects, and the most important is the quality of life (function, pain, and deformity). ^7^^,^^34^ The outcome instruments must be proven reliable, standardized, and validated to be applicable worldwide. ^35^ The generic instruments used to assess health-related quality of life (HRQL) are the SF-36 questionnaire, SRS 30, SRS 22, and the EuroQol5D instrument. ^7^ In spite of that, previous studies have reported that it is preferable to assess the condition of specific populations with certain needs. ^30^^,^^35^^,^^36^ As the SRS questionnaire and its variants could not fully portray important aspects of physical functioning such as mobility in praying for individuals with AIS, we suggest orthopedic surgeons incorporate prayer quality assessment as an adjuvant of QOL measurement in Muslim patients.

### Cobb angle correction and total fused levels effects on prayer quality changes, SRS-30, and Salah ROM

Our study found no significant nor strong correlation between Cobb angle correction and total fused level with prayer quality changes ( Table 3), quality of life (SRS-30 function and pain), and Salah ROM (bowing and prostrations) ( Table 6). To our knowledge, we are the first to identify whether curve correction and total fused levels are associated with Muslim prayer quality and Salah ROM. It seems that in the sagittal plane, the spine-pelvic-hip alignment following spinal fusion is well achieved regardless of the degree of curve correction and total fused level; hence, they are not associated with the ROM of Salah movements. Moreover, our findings are in agreement with previous studies that have reported the irrelevance of Cobb angle correction degree with postoperative SRS-30 function and pain. ^6^^,^^37^ A study by Ghandehari *et al*., revealed that the percentage of radiographic correction was positively correlated only with the total SRS-30 score (r=0.52, p<0.001) and the satisfaction domain (r=0.386, p=0.026) but not with the function and pain domains. ^6^ Likewise, Chaudhary *et al*., reported similar findings (p-values of SRS-30 function and pain correlation analyses with curve correction: 0.688 and 0.453, respectively). ^37^ Moreover, our results showed that total fused levels were not associated with function or pain. Likewise, existing literature has reported that despite resulting in better ROM, fewer fusion levels did not correlate with SRS-22 function and pain in 2-10 years of follow-up. ^2^^,^^38^^,^^39^

### Strength, limitation, and future direction

Rendering the limitation of our study in which the samples were relatively small, using patient’s perception (GPE), using a newly proposed standardized prayer movement ROM evaluation, not to mention the possibility of incorrect landmark identifying when measuring the ROM, we suggest that future studies should address these issues. Moreover, spine surgeons in largely Muslim countries should start evaluating the impact of spinal fusion on the Salah movement as part of the daily activity functional outcome. However, we believe that we are the first to assess the Islamic prayer ROM following spinal fusion surgery and attempt to identify whether spinal fusion surgery affects the Muslims’ quality of life (including their quality of prayer).

## Conclusion

Our study found that spinal fusion positively affects AIS Islamic patients in maintaining their daily Salah movement ROM and prayer quality. Orthopedic surgeons should consider incorporating prayer quality assessment as an adjuvant of the SRS-30 questionnaire to evaluate Muslim patients’ specific functional outcomes and quality of life.

## Data availability

### Extended data

Figshare: The Impact of Spinal Fusion of Adolescent Idiopathic Scoliosis in Salah (Islamic Prayer) Movement. DOI: https://doi.org/10.6084/m9.figshare.20380635. ^40^

This project contains the following underlying data:

• Appendix 1. docx (A detailed description of the AIS patients’ demographics, characteristics, and outcomes).
• Appendix 2. docx (A detailed description of demographics and characteristics of the control group (i.e., age-matched unoperated AIS patients).

Data are available under the terms of the Creative Commons Attribution 4.0 International license (CC-BY 4.0).

## References

[1] Helenius L Diarbakerli E Grauers A : Back Pain and Quality of Life after Surgical Treatment for Adolescent Idiopathic Scoliosis at 5-Year Follow-up: Comparison with Healthy Controls and Patients with Untreated Idiopathic Scoliosis. *J. Bone Jt. Surg. - Am.* 2019;101(16):1460–1466. 10.2106/JBJS.18.01370 31436653

[2] Fan H Wang Q Huang Z : Comparison of functional outcome and quality of life in patients with idiopathic scoliosis treated by spinal fusion. *Med (United States).* 2016 May;95(19):e3289. 10.1097/MD.0000000000003289 27175629 PMC4902471

[3] Weiss H-R Goodall D : Rate of complications in scoliosis surgery–a systematic review of the Pub Med literature. *Scoliosis.* 2008;3(1):1–18. 10.1186/1748-7161-3-9 18681956 PMC2525632

[4] Baldus C Bridwell K Harrast J : The scoliosis research society health-related quality of life (SRS-30) age-gender normative data: An analysis of 1346 adult subjects unaffected by scoliosis. *Spine (Phila Pa 1976).* 2011;36(14):1154–1162. 10.1097/BRS.0b013e3181fc8f98 21289576

[5] Studer D Awais A Williams N : Selective fusion in adolescent idiopathic scoliosis: a radiographic evaluation of risk factors for imbalance. *J. Child. Orthop.* 2015;9(2):153–160. 10.1007/s11832-015-0653-0 25845647 PMC4417731

[6] Ghandehari H Tari SHV Mahabadi MA : Evaluation of patient outcome and satisfaction after surgical treatment of adolescent idiopathic scoliosis using scoliosis research society-30. *Arch. Bone Jt. Surg.* 2015 Apr;3(2):109–113. 10.17795/soj-901 26110177 PMC4468625

[7] Bettany-Saltikov J Weiss HR Chockalingam N : A comparison of patient-reported outcome measures following different treatment approaches for adolescents with severe idiopathic scoliosis: A systematic review. *Asian Spine Journal. Korean Society of Spine Surgery.* 2016;10:1170–1194. 10.4184/asj.2016.10.6.1170 PMC5165010 27994796

[8] Nazish N Kalra N : Muslim Prayer- A New Form of Physical Activity: A Narrative Review. *Int. J. Heal. Sci. Res.* 2018;8(July):337–344.

[9] Ghazal K : Physical benefits of (Salah) prayer - Strengthen the faith & fitness. *J. Nov. Physiother. Rehabil.* 2018;2:043–053. 10.29328/journal.jnpr.1001020

[10] Bangash MH Alsufyani HA Karami MM : The effect of bowing and kneeling on lower back muscle. 2016;4:6–12.

[11] Safee MKM Wan Abas WAB Abu Osman NA : Activity of upper body muscles during bowing and prostration tasks in healthy subjects. *IFMBE Proc.* Springer;2011; pp.125–129. 10.1007/978-3-642-21729-6_34

[12] Puig-Diví A Escalona-Marfil C Padullés-Riu JM : Validity and reliability of the Kinovea program in obtaining angles and distances using coordinates in 4 perspectives. *PLoS One.* 2019;14(6):e0216448. 10.1371/journal.pone.0216448 31166989 PMC6550386

[13] Kamper SJ Ostelo RWJG Knol DL : Global Perceived Effect scales provided reliable assessments of health transition in people with musculoskeletal disorders, but ratings are strongly influenced by current status. *J. Clin. Epidemiol.* 2010 Jul;63(7):760–766.e1. 10.1016/j.jclinepi.2009.09.009 20056385

[14] Grech V Calleja N : WASP (Write a Scientific Paper): Parametric vs. non-parametric tests. *Early Hum. Dev.* 2018 Aug;123:48–49. 10.1016/j.earlhumdev.2018.04.014 29678516

[15] Ruxton GD Beauchamp G : Time for some a priori thinking about post hoc testing. *Behav. Ecol.* 2008 May 1;19(3):690–693. 10.1093/beheco/arn020

[16] Chen L Sun Z He J : Effectiveness and safety of surgical interventions for treating adolescent idiopathic scoliosis: A Bayesian meta-analysis. *BMC Musculoskelet. Disord.* 2020;21(1):1–15. 10.1186/s12891-020-03233-1 32615956 PMC7333422

[17] Countrymeters: Religion of the World. 2021 [cited 2021 Sep 9]. Reference Source

[18] Grim BJ Karim MS : *The Future of the Global Muslim Population: Projections for 2010-2030.* Washington D.C., USA:2011 [cited 2021 Sep 9]. Reference Source

[19] Indonesian Ministry of Religious Affairs (Kementerian Agama Indonesia): Data Umat Berdasarkan Agama (People’s data based on religion). [cited 2021 Sep 9]. Reference Source

[20] Komang-Agung IS Dwi-Purnomo SB Susilowati A : Prevalence rate of adolescent idiopathic scoliosis: Results of school-based screening in surabaya, Indonesia. *Malaysian Orthop J.* 2017;11(3):17–22. 10.5704/MOJ.1711.011 29326761 PMC5753523

[21] Djurasovic M Glassman SD Howard JM : Health-related quality of life improvements in patients undergoing lumbar spinal fusion as a revision surgery. *Spine.* 2011;36:269–276. 10.1097/BRS.0b013e3181cf1091 20739917

[22] Cho K-J Suk S-I Park S-R : Short fusion versus long fusion for degenerative lumbar scoliosis. *Eur. Spine J.* 2008;17(5):650–656. 10.1007/s00586-008-0615-z 18270753 PMC2367413

[23] Phan K Xu J Maharaj MM : Outcomes of Short Fusion versus Long Fusion for Adult Degenerative Scoliosis: A Systematic Review and Meta-analysis. *Orthop. Surg.* 2017;9(4):342–349. 10.1111/os.12357 29178306 PMC6584300

[24] Hu ZJ Fang XQ Fan SW : Iatrogenic injury to the erector spinae during posterior lumbar spine surgery: Underlying anatomical considerations, preventable root causes, and surgical tips and tricks. *Eur. J. Orthop. Surg. Traumatol.* 2014;24(2):127–135. 10.1007/s00590-012-1167-9 23417108

[25] Bastrom TP Marks MC Yaszay B : Prevalence of postoperative pain in adolescent idiopathic scoliosis and the association with preoperative pain. *Spine (Phila Pa 1976).* 2013;38(21):1848–1852. 10.1097/BRS.0b013e3182a4aa97 23883827

[26] Chidambaran V Ding L Moore DL : Predicting the pain continuum after adolescent idiopathic scoliosis surgery: A prospective cohort study. *Eur. J. Pain (United Kingdom).* 2017;21(7):1252–1265. 10.1002/ejp.1025 28346762 PMC5541247

[27] Perry M Sieberg CB Young EE : The Potential Role of Preoperative Pain, Catastrophizing, and Differential Gene Expression on Pain Outcomes after Pediatric Spinal Fusion. *Pain Manag. Nurs.* 2021;22(1):44–49. 10.1016/j.pmn.2020.05.007 32771349 PMC8742610

[28] Bailey KM Howard JJ El-Hawary R : Pain trajectories following adolescent idiopathic scoliosis correction analysis of predictors and functional outcomes. *JBJS Open Access.* 2021;6(2). 10.2106/JBJS.OA.20.00122 34056507 PMC8154436

[29] Kakar RS Li Y Brown CN : Spine and Lower Extremity Kinematics Exhibited During Running by Adolescent Idiopathic Scoliosis Patients With Spinal Fusion. *Spine Deform.* 2019;7(2):254–261. 10.1016/j.jspd.2018.08.015 30660219

[30] Holewijn RM Kingma I Kleuver M de : Spinal fusion limits upper body range of motion during gait without inducing compensatory mechanisms in adolescent idiopathic scoliosis patients. *Gait Posture.* 2017;57:1–6. 10.1016/j.gaitpost.2017.05.017 28551465

[31] Kurapati NT Krzak JJ Graf A : Effect of Surgical Fusion on Volitional Weight-Shifting in Individuals With Adolescent Idiopathic Scoliosis. *Spine Deform.* 2016;4(6):432–438. 10.1016/j.jspd.2016.08.004 27927573

[32] Delpierre Y Vernet P Surdel A : Effect of preferred walking speed on the upper body range of motion and mechanical work during gait before and after spinal fusion for patients with idiopathic scoliosis. *Clin. Biomech.* 2019;70:265–269. 10.1016/j.clinbiomech.2019.11.003 31759234

[33] Marks M Newton PO Petcharaporn M : Postoperative segmental motion of the unfused spine distal to the fusion in 100 patients with adolescent idiopathic scoliosis. *Spine.* 2012;37:826–832. 10.1097/BRS.0b013e31823b4eab 22024909

[34] Bago J Climent JM Pérez-Grueso FJS : Outcome instruments to assess scoliosis surgery. *Eur. Spine J.* 2013;22(Suppl 2):S195–S202. 10.1007/s00586-012-2352-6 22576158 PMC3616464

[35] Du C Yu J Zhang J : Relevant areas of functioning in people with adolescent idiopathic scoliosis on the international classification of functioning, disability and health: The patients’ perspective. *J. Rehabil. Med.* 2016;48(9):806–814. 10.2340/16501977-2147 27711934

[36] Alamrani S Rushton A Gardner A : Outcome measures evaluating physical functioning and their measurement properties in adolescent idiopathic scoliosis: A protocol for a systematic review. *BMJ Open.* 2020;10(4). 10.1136/bmjopen-2019-034286 32241788 PMC7170637

[37] Chaudhary RK Kaucha D Banskota B : Correlation between Radiological Outcome and Health Related Quality of Life after Posterior Spinal Fusion for Adolescent Idiopathic Scoliosis. *J. Nepal Health Res. Counc.* 2021;19(1):44–47. 10.33314/jnhrc.v19i1.3245 33934131

[38] Uehara M Takahashi J Ikegami S : Correlation of Lower Instrumented Vertebra with Spinal Mobility and Health-related Quality of Life after Posterior Spinal Fusion for Adolescent Idiopathic Scoliosis. *Clin. Spine Surg.* 2019;32(7):E326–E329. 10.1097/BSD.0000000000000794 31361270

[39] Ohashi M Bastrom TP Marks MC : The Benefits of Sparing Lumbar Motion Segments in Spinal Fusion for Adolescent Idiopathic Scoliosis Are Evident at 10 Years Postoperatively. *Spine.* 2020;45:755–763. 10.1097/BRS.0000000000003373 31923128

[40] Irianto KA Firas NR Binti CGS : The Impact of Spinal Fusion of Adolescent Idiopathic Scoliosis in Salah (Islamic Prayer) Movement. Extended Dataset: figshare. 2022. 10.6084/m9.figshare.20380635.v1 Reference Source PMC11099510 38765242

