# Supplementary material for: The impact of spinal fusion of adolescent idiopathic scoliosis in Salah (Islamic Prayer) movement: a retrospective case-control study
Source: F1000Res. 2024 Jun 24;11:1054. Originally published 2022 Sep 15. [Version 3] doi: 10.12688/f1000research.124255.3 (PMC11099510; doi:10.12688/f1000research.124255.3)
Supplement: Peer review history

# Reviewer report: Reviewer response for version 3

**DOI:** 10.5256/f1000research.167801.r294866

**Published:** 2024-08-07

**Authors:** Vibhu Krishnan Viswanathan

**Affiliations:** 1 Rela Institute and Medical Center, Chennai, India

We congratulate the authors for their hard work. 

 The recommended changes have been incorporated and the manuscript may be accepted for indexing in the current form.

Is the work clearly and accurately presented and does it cite the current literature?

Partly

If applicable, is the statistical analysis and its interpretation appropriate?

Partly

Are all the source data underlying the results available to ensure full reproducibility?

Partly

Is the study design appropriate and is the work technically sound?

Yes

Are the conclusions drawn adequately supported by the results?

Partly

Are sufficient details of methods and analysis provided to allow replication by others?

Partly

Reviewer Expertise:

spine surgery

I confirm that I have read this submission and believe that I have an appropriate level of expertise to confirm that it is of an acceptable scientific standard.

# Reviewer report: Reviewer response for version 2

**DOI:** 10.5256/f1000research.145117.r249118

**Published:** 2024-05-16

**Authors:** Hiva Razavi

**Affiliations:** 1 Department of Biomechanics, University of Nebraska at Omaha, Omaha, NE, USA

1. While the research question is clearly defined, explicitly stating a hypothesis would specify the expected effect of spinal fusion on Salah prayer movements and guide the study's design and statistical analysis more effectively.

 2. The study could be strengthened by detailing specific measures undertaken to mitigate potential sources of bias, such as selection bias and measurement bias. This includes strategies like blinding, matching cases and controls, or adjusting for confounders, which are crucial for enhancing the validity and reliability of the findings.

 3. A more comprehensive discussion on how the study's limitations might affect the findings would lend greater credibility to the research. Understanding these limitations' potential direction and magnitude would allow for a more balanced interpretation of the results.

 4. Including a justification for determining study size regarding considerations for statistical power and expected effect size is vital. This would provide critical insights into the study's capacity to detect meaningful differences, thereby affirming the robustness of its conclusions.

Is the work clearly and accurately presented and does it cite the current literature?

Yes

If applicable, is the statistical analysis and its interpretation appropriate?

Yes

Are all the source data underlying the results available to ensure full reproducibility?

Yes

Is the study design appropriate and is the work technically sound?

Yes

Are the conclusions drawn adequately supported by the results?

Yes

Are sufficient details of methods and analysis provided to allow replication by others?

Yes

Reviewer Expertise:

Scoliosis, Biomechanics

I confirm that I have read this submission and believe that I have an appropriate level of expertise to confirm that it is of an acceptable scientific standard.

# Response

**Published:** 2024-06-10

**Authors:** komang irianto

**Affiliations:** Airlangga University, Indonesia

We thank the reviewer for their input. We have amended our manuscript accordingly. Please find the details below:

1. Hypothesis: A hypothesis has been added at the end of the introduction section.
2. Bias: Thank you for your input. As the nature of this study design, i.e., retrospective case-control study, our study is inevitable from the potential of such biases. Hence, we acknowledge our limitations in the last part of our Discussion section.
3. Study limitations: We thank the reviewer for their input. We have added a new paragraph to provide an in-depth discussion of our study limitations and their impacts on our results.
4. Study size: Thank you for your valuable feedback. We acknowledge that an a priori sample size calculation was not performed for this retrospective case-control study, as we utilized all available patient data from our records. However, we recognize that this approach may limit the statistical power of our findings. Hence, we have added a new explanation of this issue in the study limitations section.

# Reviewer report: Reviewer response for version 2

**DOI:** 10.5256/f1000research.145117.r227784

**Published:** 2024-05-16

**Authors:** Vibhu Krishnan Viswanathan

**Affiliations:** 1 Rela Institute and Medical Center, Chennai, India

Title and abstract: Please add study design clearly - Prospective or Retrospective.

 Introduction: Very long. Since the focus is primarily on ROM, the relevant literature on ROM alone may be discussed.

 Methods: Well explained.

 Results: Relatively small sample size. Please present all relevant p values.

 Tables may be more clearly presented. In the current form, they are not very clear

 Discussion: There are some grammatical and typographical errors. Overall well discussed.

 Additional literature on similar studies regarding spinal ROM after AIS surgeries may be discussed.

Is the work clearly and accurately presented and does it cite the current literature?

Partly

If applicable, is the statistical analysis and its interpretation appropriate?

Partly

Are all the source data underlying the results available to ensure full reproducibility?

Partly

Is the study design appropriate and is the work technically sound?

Yes

Are the conclusions drawn adequately supported by the results?

Partly

Are sufficient details of methods and analysis provided to allow replication by others?

Partly

Reviewer Expertise:

spine surgery

I confirm that I have read this submission and believe that I have an appropriate level of expertise to confirm that it is of an acceptable scientific standard, however I have significant reservations, as outlined above.

# Response

**Published:** 2024-06-10

**Authors:** komang irianto

**Affiliations:** Airlangga University, Indonesia

We thank the reviewer for their input. We have amended our manuscript accordingly. Please find the details below:

1. Title and abstract: We have amended our title and abstract as per your suggestion; the word “retrospective” has been added.
2. Introduction: Thank you for your kind review. The introduction has been shortened whilst still focusing on ROM; a new reference has been added to support this.
3. Results: All relevant p-values have been added to the text and tables.
4. Discussion: Thank you for your input. We have addressed the problem with grammatical and typographical errors. We also added a new paragraph with relevant literature on spinal ROM after AIS surgeries.

# Reviewer report: Reviewer response for version 1

**DOI:** 10.5256/f1000research.136441.r156368

**Published:** 2023-02-20

**Authors:** Yasser Allam

**Affiliations:** 1 Waldklinikum Gera, Gera, Germany; 2 Department of Orthopaedic Surgery and Traumatology Faculty of Medicine, Alexandria University, Alexandria, Egypt

1. It would be interesting to include the whole 5 items of SRS 30 in this study and not only pain and function.
2. Control group degree of scoliosis is less than study material. It would be better if the control group has nearly the same degree of scoliosis as patient group.
3. In Fig. 3, it was mentioned that the patient had got 50° scoliosis and in Fig. 4, 45° scoliosis which from my point of view can be treated conservative. what were the measures for patient selection i.e. degree of scoliosis
4. I would prefer to write muscle fibrosis instead of muscle degeneration (4th paragraph in discussion) 

Is the work clearly and accurately presented and does it cite the current literature?

Yes

If applicable, is the statistical analysis and its interpretation appropriate?

Yes

Are all the source data underlying the results available to ensure full reproducibility?

Yes

Is the study design appropriate and is the work technically sound?

Yes

Are the conclusions drawn adequately supported by the results?

Yes

Are sufficient details of methods and analysis provided to allow replication by others?

Yes

Reviewer Expertise:

spine surgery

I confirm that I have read this submission and believe that I have an appropriate level of expertise to confirm that it is of an acceptable scientific standard.

# Response

**Published:** 2023-03-03

**Authors:** komang irianto

**Affiliations:** Airlangga University, Indonesia

Dear Dr. Yasser Allam,

 Thank you for your kind review of this manuscript, which has been very helpful in improving the manuscript. We also thank the reviewer for the careful reading of our text. Here we present our reply to each suggestion separately as the following:

*1. "It would be interesting to include the whole 5 items of SRS-30 in this study and not only pain and function."*

**Answer: **We only utilized two of five assessment categories in the SRS-30 questionnaire (pain, function or activity, self-image, mental health, and satisfaction with management). The self-image, mental health, and satisfaction items are not directly relevant to our study. This study focuses on the daily Islamic prayer function (Salah activity), which needs a series of complex movements involving the spine, hip, and knee. The prayer activity itself is believed ought to be performed with the correct movements as mandated by their religion within the correct timeframe (5 times a day at certain hours), which makes patients worry if they cannot perform it after corrective surgery. Hence, we deem the two chosen items (pain and function) to be the two most critical and relevant tools to evaluate patients’ quality of life relating to their ability to fulfill their religious obligation.

 However, we acknowledge the reviewer’s suggestion as our limitation. We have added the reviewer’s suggestion in our limitation section:

 “Another drawback is that we only assessed two items of the SRS-30 questionnaire (pain and function), as we deem the most critical evaluation tools for performing the Islamic Salah prayer activity. Future studies should attempt to assess all five items of SRS-30 for a more comprehensive evaluation.”

*2. "The control group's degree of scoliosis is less than the study material. It would be better if the control group has nearly the same degree of scoliosis as the patient group."*

**Answer: **We thank the reviewer for their comment. We also realized this as the limitation of this study, and we have previously justified this in the Results section, paragraph 2, as follows:

 “Although the preoperative Cobb angles between the two groups differed significantly (67.1±16.2° and 52.3±14.0°, respectively), it does not necessarily reflect a non-homogeneity between them. The nature of this study is to compare the operated and unoperated patients. Those who underwent surgery would generally have a higher degree of Cobb angle, though the clinical significance of 14.9° mean difference is debatable.”

 However, based on the reviewer’s suggestion, we have added this in our limitation section as follows:

 “Moreover, due to the nature of this study, the control group had a lesser Cobb angle value. This difference is unavoidable because the larger curves group would most unequivocally be offered surgery rather than conservative treatment; hence, this condition has become a natural limitation of this study.”

*3. "In Fig. 3, it was mentioned that the patient had got 50° scoliosis, and in Fig. 4, 45° scoliosis which from my point of view can be treated conservatively. what were the measures for patient selection i.e. degree of scoliosis"*

**Answer: **We thank the reviewer for their meticulous review. There are several considerations for the treatment choice (explained below), which is specific for each patient. We have added our surgery indication in the Method section:

 “As for the surgical indication, we follow the indication outlined by the Scoliosis Research Society (SRS), namely when the curves exceed 45°-50°. Patients with less than 45° curvature are generally treated with conservative treatment, unless in skeletally immature patients who are at high risk of developing progressive deformity. In the “gray zone” cases (i.e., 45°-50°), the clinical complaints (pain, diminished capacity for activities) along with psychological state play a major role in determining the treatment choice. However, other contributing factors, such as the patient’s socioeconomic condition, willingness to undergo a surgical procedure, and family support, may also affect the final decision of treatment choice.”

*4."I would prefer to write muscle fibrosis instead of muscle degeneration (4th paragraph in discussion)"*

**Answer**: We agree with your opinion. We have replaced the degeneration with "fibrosis".
